# Oxidative Stress and DNA Damage in *Pagrus major* by the Dinoflagellate *Karenia mikimotoi*

**DOI:** 10.3390/toxins15100620

**Published:** 2023-10-19

**Authors:** Yun Kyung Shin, Do Yeon Seo, Hye-Jin Eom, Mira Park, Minji Lee, Young-Eun Choi, Young-Seok Han, Jae-Sung Rhee, Youn-Jung Kim

**Affiliations:** 1 National Institute of Fisheries Science, Busan 46083, Republic of Korea; yunkshin@korea.kr; 2 Department of Marine Science, College of Natural Sciences, Incheon National University, Incheon 22012, Republic of Korea; ehdus_spdlqj@naver.com (D.Y.S.); ksks80@naver.com (H.-J.E.); youngchoi95@naver.com (Y.-E.C.); 3 Risk Assessment Division, Environmental Health Research Department, National Institute of Environmental Research, Incheon 22012, Republic of Korea; 4 Research Institute of Basic Sciences, Incheon National University, Incheon 22012, Republic of Korea; mira0295@gmail.com; 5 South Sea Fisheries Research Institute, National Institute of Fisheries Science, Yeosu 59780, Republic of Korea; minzlee@korea.kr; 6 Eco Sustainable Solution Center Korea Conformity Laboratories, Incheon 40684, Republic of Korea; 7 Neo Environmental Business Co., Bucheon 14523, Republic of Korea; han-kong@daum.net; 8 Yellow Sea Research Institute, Incheon 22012, Republic of Korea

**Keywords:** *Karenia mikimotoi*, *Pagrus major*, oxidative stress, DNA damage

## Abstract

*Karenia mikimotoi* is a common species of red tide dinoflagellate that causes the mass mortality of marine fauna in coastal waters of Republic of Korea. Despite continuous studies on the ecophysiology and toxicity of *K. mikimotoi*, the underlying molecular mechanisms remain poorly understood. Red sea bream, *Pagrus major,* is a high-value aquaculture fish species, and the coastal aquaculture industry of red sea bream has been increasingly affected by red tides. To investigate the potential oxidative effects of *K. mikimotoi* on *P. major* and the molecular mechanisms involved, we exposed the fish to varying concentrations of *K. mikimotoi* and evaluated its toxicity. Our results showed that exposure to *K. mikimotoi* led to an accumulation of reactive oxygen species (ROS) and oxidative DNA damage in the gill tissue of *P. major*. Furthermore, we found that *K. mikimotoi* induced the activation of antioxidant enzymes, such as superoxide dismutase, catalase, glutathione peroxidase, and glutathione reductase, in the gill tissue of *P. major*, with a significant increase in activity at concentrations above 5000 cells/mL. However, the activity of glutathione *S*-transferase did not significantly increase at the equivalent concentration. Our study confirms that oxidative stress and DNA damage is induced by acute exposure to *K. mikimotoi,* as it produces ROS and hypoxic conditions in *P. major*. In addition, it was confirmed that gill and blood samples can be used as biomarkers to detect the degree of oxidative stress in fish. These findings have important implications for the aquaculture of red sea bream, particularly in the face of red tide disasters.

## 1. Introduction

The planktonic microalgae of the world’s oceans are the primary producers of marine ecosystems and are an important food source for filter-feeding shellfish (oysters, mussels, and clams), crustaceans, and fishes [1]. As the coastal environment continues to change, owing to the increase in human activities in coastal waters as well as climate change, the occurrence of red tides has been increasing [2,3]. This indicates that the degree of damage caused by aquatic organisms is likely to increase [4]. The occurrence of red tides is caused by the excessive growth of dinoflagellates, diatoms, raphidophytes, or other taxonomic groups when environmental conditions favoring their blooms are established [5]. Certain red tide-causing algae produce hemolytic toxins that dissolve fish gills, or ichthyotoxins that induce the production of reactive oxygen species (ROS) [6,7]. Other negative effects, such as anoxia, hypoxia, or irradiance reduction, can be induced by an increase in the number of phytoplankton on the seawater surface [8]. This results in serious damage, negatively impacting aquaculture, humans, and the environment owing to the massive loss of marine life [9,10].

Red sea bream, a major aquaculture species on the southern coast of Korea, is a value-added aquatic species. Not only is its domestic demand increasing, but it is also exported as live fish, which contributes to Republic of Korea’s economy [11]. The production of marine fish has been increasing, primarily because of marine enclosure aquaculture on the southern coast of Republic of Korea. According to the Korean Statistical Information Service (KOSIS), 7,205,511 (97.8%) of 73,675,000 national red sea breams are located on the southern coast. The vast majority of red sea bream is produced in the coastal areas of Republic of Korea. Because red sea bream is cultured via the enclosure aquaculture method, it is directly exposed to the effects of natural environmental changes such as red tide occurrence and temperature change, unlike land-based culture, which involves cultivation inside the facility [12].

The number of reports and inquiries related to aquaculture damage on the southern coast of Republic of Korea has increased every year, especially since 2011. Red tide status data from 2014 to 2017 were obtained from the monitoring program provided by the National Fisheries Research and Development Institute (NFRDI) (Table 1). In an analysis of the causes of mass mortality of fish cultured on the southern coast of Republic of Korea, harmful algal blooms (HABs) accounted for the largest portion (37.4%), followed by high temperatures (31%) and low temperatures (26.5%) [13]. During the 1970s and 1980s, the dominant HAB species was dinoflagellate *Karenia mikimotoi*. However, in the 1990s and the 2000s, dinoflagellates *Cochlodinium polykrikoides* and *Ceratium* spp. and raphidophyte *Chattonella* spp. emerged as the primary HAB species [14]. Recently, red tides caused by *Karenia* have been reported to increase in Japan [15], but have not occurred in Republic of Korea since 2016 (Table 1). In the case of *Karenia*, HABs are more often caused by thermophilic and photochromic species than they are caused by *Cochlodinium*. As the temperature of the sea surface has increased recently, the environment favored by *Karenia* has been established [16]. In addition, owing to the low-salinity phenomenon, the proliferation of other competing organisms has been restricted, and favorable conditions for the growth of *Karenia* have formed.

*Karenia mikimotoi* (*K. mikimotoi*) is the most common red tide dinoflagellate worldwide. *K. mikimotoi* blooms have been reported in the western part of Seto-Inland Sea, Japan [17], in Hakodate Bay, Japan [18], in eastern Chinese waters [19], along the Hong Kong coast [20], in western Irish waters [21], along the American East Coast, and along the European coast [22]. *K. mikimotoi* blooms caused severe damage to fisheries in 1981 for the first time in Korea [23]. Recently, *K. mikimotoi* caused red tides, resulting in damage, and further studies on this species are needed. Previous studies on *K. mikimotoi* have focused on its physiology, ecological life history [24], the impact of environmental stress [25,26], the production of ROS [7,27,28,29], and toxicity [30]. However, there is a lack of studies focusing on the death-inducing mechanism and mortality of domestic aquaculture organisms. In fish, the toxic effect of *K. mikimotoi* on larval behavior and gill tissue was analyzed [31,32]. In addition to finfish, *K. mikimotoi* is toxic to various marine organisms such as abalone [33,34], benthic organisms such as blue mussels, urchins, starfish [35], and copepod *Tigriopus japonicus* [29], and rotifer *Brachionus plicatilis* [36], all of which die when exposed to high concentrations of *K. mikimotoi*. In addition, it has also been reported that the exposure of MeOH/H_2_O extract of *K. mikimotoi* to mammalian cells affects their proliferation and morphology [37].

In this study, we measured the toxicity and potential oxidative effects of various concentrations of *K. mikimotoi* on the gill tissues or blood of *P. major*. To minimize the potential influence of additional or combined unknown toxins that might be produced by *K. mikimotoi*, the fish were subjected to acute exposure using harvested *K. mikimotoi*. This harvested *K. mikimotoi* was centrifuged and promptly dissolved in a fresh medium. Previous studies have indicated that *K. mikimotoi* does not contain shellfish poisons [38,39], brevetoxins [16], or karlotoxin [40]. To observe the disturbance in redox homeostasis induced by *K. mikimotoi*, we measured the intracellular contents of malondialdehyde (MDA) and glutathione (GSH) via an analysis of the enzymatic activity of the antioxidant defense system. The level of DNA damage was confirmed using comet analysis. Our findings can aid in comprehending the potential impact of *K. mikimotoi* on fish gills and their response to HABs, as well as provide valuable information on the health of aquatic organisms during red tides. This study’s results can also be beneficial for red tide management.

## 2. Results

### 2.1. Acute Toxicity

No mortality of juvenile *P. major* was observed at any concentration (1000–7000 cells/mL) of *K. mikimotoi* during a 24 h exposure period and within a depuration time of 3 h.

### 2.2. MDA Content as an Oxidative Index

**Figure 1:**
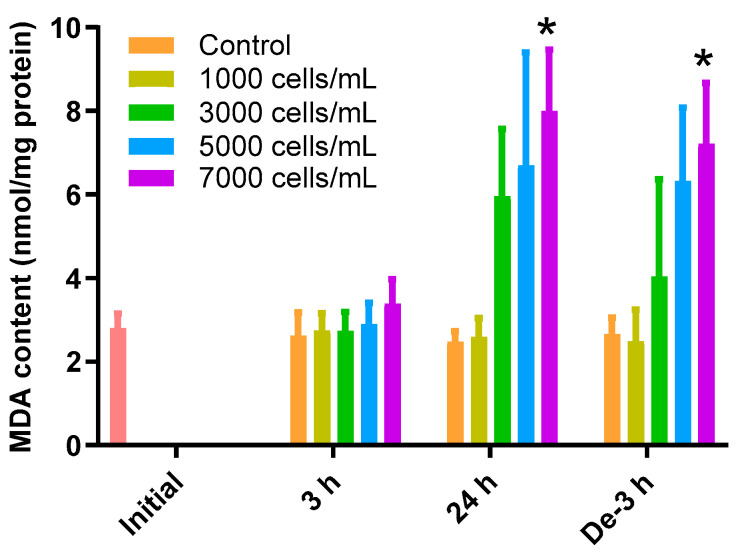
Effects of different concentrations of *K. mikimotoi* (1000, 3000, 5000, and 7000 cells/mL) on MDA levels in the gill tissues of *P. major* after exposure for 3 h and 24 h, and depuration for 3 h. All statistical data are expressed as mean ± standard deviation. Statistical significance is indicated by an asterisk (*) in comparison with the control (*p* < 0.05). MDA, malondialdehyde; Initial, 0 h of exposure; 3 h, 3 h of exposure; 24 h, 24 h of exposure; De-3 h, 3 h of depuration.

The MDA content in the gill tissue increased depending on the concentration of *K. mikimotoi* during the 24 h exposure and depuration time (Figure 1). During the 3 h exposure, there was no significant difference in the change of MDA in the gill tissues of all treatment groups compared to that of the control. In contrast, exposure to 7000 cells/mL (8.00 ± 1.47 nmol/mg) of *K. mikimotoi* resulted in significantly higher levels of MDA (*p* < 0.05) after exposure for 24 h compared to that of the control (2.48 ± 0.24 nmol/mg). In the gill tissues with a depuration period of 3 h after exposure to 7000 (7.21 ± 1.45 nmol/mg) cells/mL of *K. mikimotoi*, MDA levels were still significantly increased compared to that of the control (2.66 ± 0.39 nmol/mg).

### 2.3. ROS Scavenging Enzymes Activities

**Figure 2:**
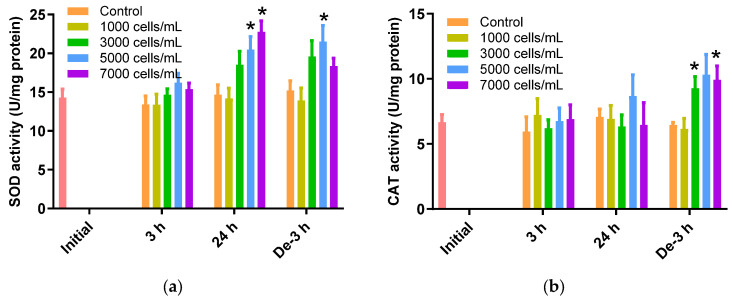
Effects of different concentrations of *K. mikimotoi* (1000, 3000, 5000, and 7000 cells/mL) on the enzymatic activities of (**a**) SOD and (**b**) CAT in the gill tissues of *P. major* after exposure for 3 or 24 h and depuration for 3 h. All statistical data are expressed as mean ± standard deviation. Statistical significance is indicated by an asterisk (*) in comparison with the control (*p* < 0.05). SOD, superoxide dismutase; CAT, catalase; Initial, 0 h of exposure; 3 h, 3 h of exposure; 24 h, 24 h of exposure; De-3 h, 3 h of depuration.

The SOD activity in the 24 h exposure and 3 h depuration groups increased in response to the *K. mikimotoi* concentration increase (Figure 2a). A significant increase of 20.46 ± 1.70 U/mg and 22.76 ± 1.42 U/mg was noted for 5000 cells/mL and 7000 cells/mL of *K. mikimotoi*-exposed *P. major*, respectively, for 24 h compared to the control (14.65 ± 1.28 U/mg) (*p* < 0.05). After 3 h of depuration, SOD activity was significantly increased when exposed to concentrations of 5000 cells/mL of *K. mikimotoi* (21.48 ± 2.08 U/mg) (Figure 2a).

The CAT activity in gill tissues of *P. major* treated with *K. mikimotoi* for 3 h and 24 h was not significantly elevated, whereas an increase in activity was observed at 3000 and 7000 cells/mL after 3 h of depuration (3000 cells/mL: 9.28 ± 0.89 U/mgand 7000 cells/mL: 9.92 ± 1.07 U/mg) compared to that of the control (6.45 ± 0.21 U/mg) (Figure 2b).

### 2.4. Glutathione Dependent Metabolic Pathway 

**Figure 3:**
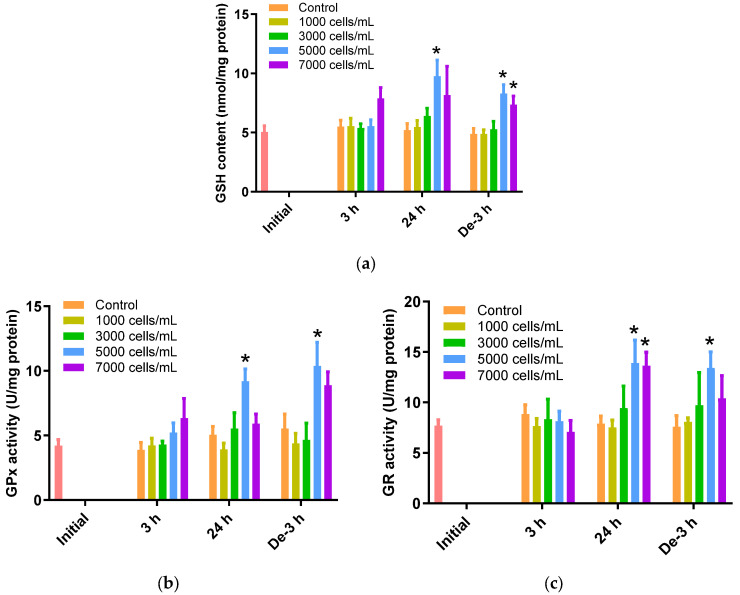
Effects of different concentrations of *K. mikimotoi* (1000, 3000, 5000, and 7000 cells/mL) on enzymatic activities of (**a**) GSH, (**b**) GPx, and (**c**) GR in the gill tissue of *P. major* after exposure for 3 or 24 h and depuration for 3 h. All statistical data are expressed as mean ± standard deviation. Statistical significance is indicated by an asterisk (*) compared with the control (*p* < 0.05). GSH, glutathione; GPx, glutathione peroxidase; GR, glutathione reductase; Initial, 0 h of exposure; 3 h, 3 h of exposure; 24 h, 24 h of exposure; De-3 h, 3 h of depuration.

The gill tissues of *P. major* showed a noteworthy rise in GSH levels when exposed to 5000 cells/mL of *K. mikimotoi* for 24 h (9.75 ± 1.36 nmol/mg) (*p* < 0.05). However, gill tissues after exposure to 1000 and 3000 cells/mL of *K. mikimotoi* showed no significant changes, regardless of time. After 3 h of depuration, GSH levels were still higher after exposure to 5000 (8.30 ± 0.74 nmol/mg) and 7000 (7.36 ± 0.73 nmol/mg) cells/mL of *K. mikimotoi* compared to those of the control, but were decreased compared with those of the gills exposed for 24 h (Figure 3a).

GPx activity was significantly increased after exposure to 5000 cells/mL of *K. mikimotoi* for 24 h (9.20 ± 0.95 U/mg) (*p* < 0.05). In addition, GPx activity was increased in gill tissues exposed to 5000 cells/mL (10.38 ± 1.83 U/mg) of *K. mikimotoi* for 3 h of depuration (Figure 3b). In this study, GPx was increased in the gill tissue of *P. major* exposed to high concentrations of *K. mikimotoi* but did not decrease after 3 h of depuration. These results suggest that the activation mechanism of GPx decreases as GSH decreases due to a defense mechanism.

When exposed to 5000 cells/mL of *K. mikimotoi*, significantly higher levels of GR activity were observed after exposure for 24 h (13.91 ± 2.26 U/mg) and depuration for 3 h (13.40 ± 1.61 U/mg) (*p* < 0.05). Additionally, significant differences were observed only after 24 h exposure to 7000 cells/mL of *K. mikimotoi* (Figure 3c).

### 2.5. GST Activity as a Detoxification Enzyme 

**Figure 4:**
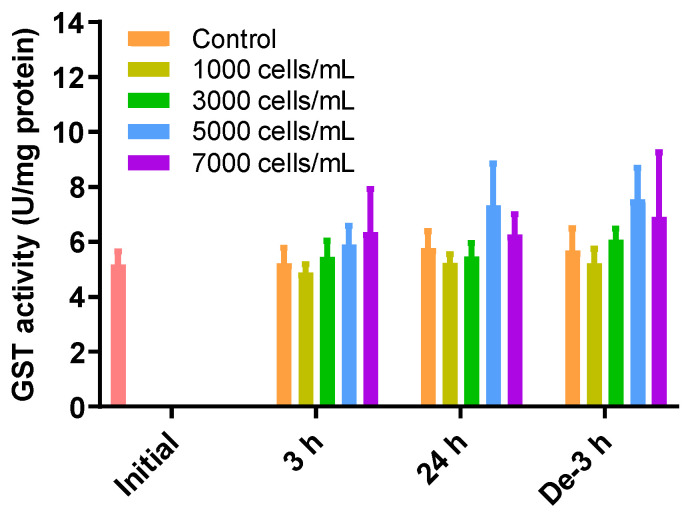
Effects of different concentrations of *K. mikimotoi* (1000, 3000, 5000, and 7000 cells/mL) on enzymatic activities of GST in the gill tissue of *P. major* after exposure for 3 or 24 h and depuration for 3 h. All statistical data are expressed as mean ± standard deviation. GST, glutathione S-transferase; Initial, 0 h of exposure; 3 h, 3 h of exposure; 24 h, 24 h of exposure; De-3 h, 3 h of depuration.

GSH can also be removed via GSH-induced inclusion and conjugation with GST. However, there were no significant differences in the levels of GST activity between all treatments and depuration times and all treatment concentrations (Figure 4).

### 2.6. DNA Damage in Blood Samples of P. major

**Figure 5:**
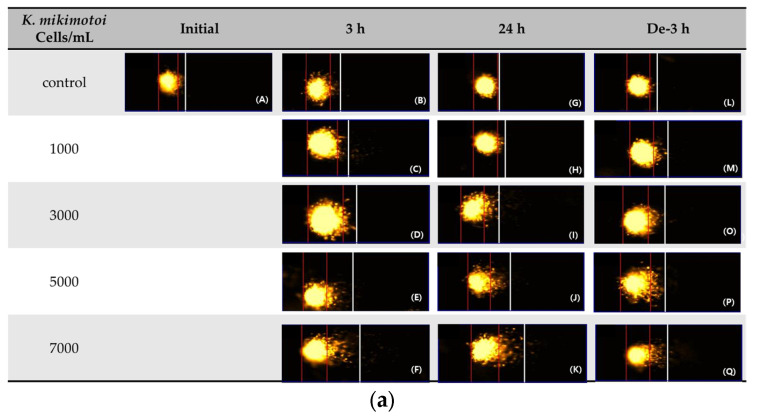

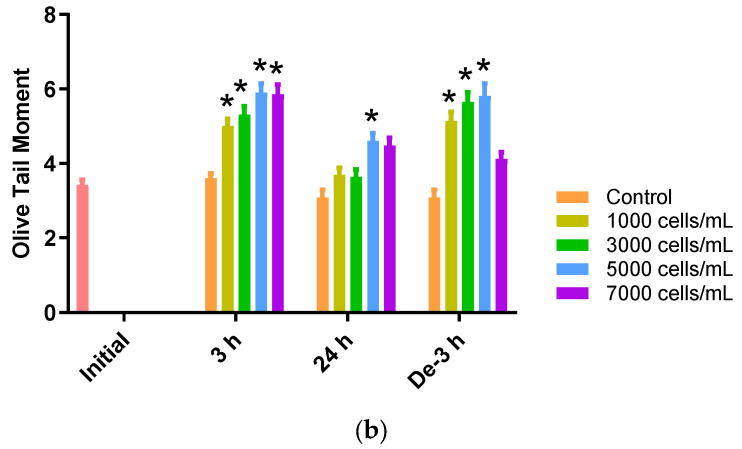
Effects of different concentrations of *K. mikimotoi* (1000, 3000, 5000, and 7000 cells/mL) on DNA damage indicator. (**a**) Nucleus comet images; (A) represents the comet image of the initial control group. The comet images corresponding to the concentrations of control, 1000, 3000, 5000, and 7000 cells/mL are shown in (B–F) for groups exposed for 3 h, (G–K) for groups exposed for 24 h, and (L–Q) represent groups that underwent depuration for 3 h. (**b**) Olive tail moments (OTMs) in the bloods of *P. major* after exposure for 3 h and 24 h, or depuration for 3 h. All statistical data are expressed as mean ± standard deviation. Statistical significance is indicated by an asterisk (*) compared with the control (*p* < 0.05). Initial, 0 h of exposure; 3 h, 3 h of exposure; 24 h, 24 h of exposure; De-3 h, depuration for 3 h.

The comet assay (or single-cell gel electrophoresis (SCGE)) can be used to assess the environmental impact of genotoxic materials in aquatic organisms. DNA damage in the blood samples from *P. major* exposed to different concentrations of *K. mikimotoi* was expressed as the Olive tail moment (OTM). We observed that the levels of the OTM increased significantly at all treatment concentrations of *K. mikimotoi* under 3 h of exposure and depuration for 3 h (except 7000 cells). After 24 h of exposure, the OTM was significantly increased only after exposure to 5000 cells of *K. mikimotoi* (Figure 5).

### 2.7. Correlation Analysis

**Figure 6:**
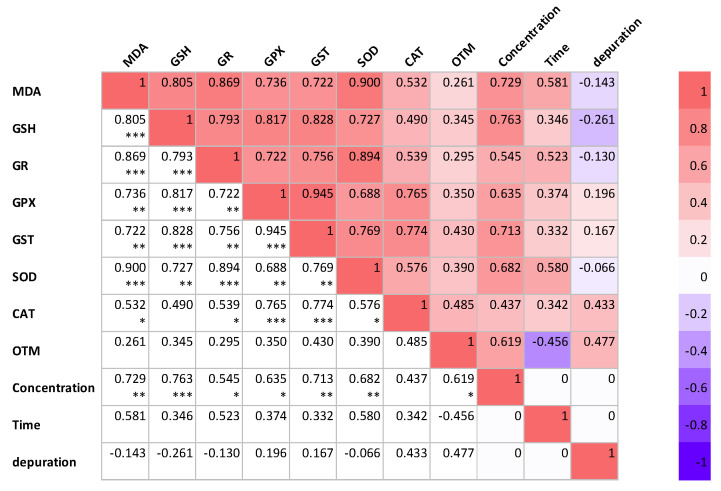
Correlations among various variables investigated in the gills and blood of *P. major* at various concentrations and times, and under the depuration of *K. mikimotoi*. The values presented are correlation coefficients (r). * Correlation is significant at the 0.05 level (2−tailed). ** Correlation is significant at the 0.01 level (2−tailed). *** Correlation is significant at the 0.005 level (2−tailed).

Figure 6 illustrates the Pearson correlation among different variables in *P. major* under experimental conditions. The figure also depicts the interaction between the three experimental factors (concentration, treatment time, and the depuration of *K. mikimotoi*) for all the parameters investigated in this study. When correlation analysis was conducted with oxidative stress markers, the response was dependent on the exposure concentration of *K. mikimotoi*, but was independent of the exposure time and depuration of *K. mikimotoi*. The response of MDA content was positively correlated (*p* < 0.05–0.001) with the exposure concentration of *K. mikimotoi* in the gills of *P. major*. Furthermore, our study revealed that the OTM measured in the blood was weakly correlated with the oxidative stress biomarker measured in the gills. These findings demonstrate that oxidative stress biomarkers can be used to determine the exposure levels of *K. mikimotoi* in *P. major*.

## 3. Discussion

Previous studies have reported that the toxicity of *K. mikimotoi* in fish is species-specific. According to Ou (2006), trout, salmon, and flounder all died within a short time due to severe gill damage [41]. However, the red sea bream used in this study exhibited a slightly different trend. No lethality was observed at a certain concentration of *K. mikimotoi* (>1000 cell/mL), but oxidative stress and DNA damage were observed, though at the same time, the expression of antioxidant enzymes also increased. This defense mechanism is thought to be responsible for the survival of red sea bream.

Oxidative stress occurs when there is an imbalance in the ratio of biological oxidants to antioxidants, and can lead to oxidative damage to lipids, proteins, carbohydrates, and nucleic acids. Excessive ROS production is considered an important indicator of oxidative damage [42]. Previous studies have shown that *K. mikimotoi* produces ROS, and significant levels of O_2_^−^ and H_2_O_2_ were also detected [30,43]. In addition, *K. mikimotoi* attaches to the gill tissue of red sea bream to interfere with respiration, which can lead to ROS due to hypoxia. The concentrations of *K. mikimotoi* used in this experiment (>1000 cell/mL) could induce hypoxia to an amount sufficient to lower dissolved oxygen levels [8]. It is therefore unclear whether the results obtained in this experiment are due to either the exogenous ROS produced by *K. mikimotoi* itself or the intracellular ROS produced directly by the gill tissue interfering with the respiration of the sea bream. 

Organisms have evolved their own defense mechanisms to protect themselves from oxidative stress, such as hypoxia. In fish, an increase in the levels of MDA and antioxidant enzymes can be interpreted as a defense mechanism to combat oxidative stress [43,44,45]. However, excessive MDA can damage cell structure and function [46]. For instance, a previous study showed that the MDA content was significantly increased in the mammalian cell lines exposed to the polar lipid extracts of *K. mikimotoi* [37]. The increased MDA levels during the depuration period indicate a persistent detrimental effect of previous exposure to *K. mikimotoi*, even though the water has been completely depurated (Figure 1). This result also suggests that a 3 h depuration period is insufficient to eliminate the presence of MDA. GSH is a free radical-scavenging antioxidant and an important indicator of an organism’s detoxification ability [47]. Similar to a previous study on *P. major* exposed to high concentrations of selenium, which induces oxidative stress, the present study observed an increase in GSH levels in the liver and gill tissues of red sea bream [48]. However, in this study, the GSH concentration decreased with an increasing exposure time, which is considered to be due to continuous redox reactions catalyzed via GPx and GR activities.

SOD and CAT are crucial enzymes for the removal of ROS in a cell. The induction of these enzymes is one of the defense mechanisms against oxidative damage (Figure 2a,b). SOD converts the highly reactive superoxide radical O_2_^−^ into H_2_O_2_. The activity of SOD increases with increasing concentrations of superoxide anions derived from algal blooms [49]. CAT neutralizes H_2_O_2_ by converting it into water and molecular oxygen. However, the decrease in CAT activity after 24 h of exposure to *K. mikimotoi* may be due to a decrease in the rate of the reaction as a result of the excessive production of H_2_O_2_. The inhibition of CAT by superoxide radicals has been reported in previous studies of freshwater fish [50,51]. GPx decomposes H_2_O_2_ into H_2_O and O_2_. GPx requires GSH to function as an antioxidant enzyme in the redox cycle, which is required for H_2_O_2_ decomposition [52]. Therefore, the correlation between GSH and GPx was very high, and similar results were obtained in the present study. GR plays a role in generating GSH by reducing glutathione disulfide (GSSG), which is generated by the antioxidant defense system.

ROS produced by *K. mikimotoi* induced DNA damage and lipid peroxidation in the form of H_2_O_2_, an O_2_^−^ form, or a product of SOD. Three defense mechanisms are used to protect cells from oxidative stress (Figure 5). In this experiment, the levels of GPx and GR enzymes involved in the GPx cycle were greatly increased (Figure 3b,c), and the level of GSH, which is an intermediate product, also increased (Figure 3a). In addition, CAT activity increased during the CAT cycle. The GPx and CAT cycle were used to remove the generated H_2_O_2_. Taken together, the overall results suggest that oxidative homeostasis is directly affected by acute *K. mikimotoi* exposure in this species. The inconsistencies observed in the enzymatic activities of SOD and CAT can be explained by their sequential relationships. While both enzymes are part of the first-line antioxidant defense against ROS, CAT catalyzes the degradation or reduction of H_2_O_2_, which is the final product of the dismutation of the superoxide anion conducted by SOD [53]. Therefore, conducting a more detailed time-course analysis could be helpful for understanding the entire defense strategy, as the results were only analyzed at three time points during acute exposure.

Furthermore, the conjugation reaction by GST did not change significantly, even when the concentration of *K. mikimotoi* was increased (Figure 4). Since GST, as one of the important phase II detoxification enzymes, mediates GSH conjugation with xenobiotics to neutralize and excrete them, it is possible that *K. mikimotoi*-triggered oxidative damage is not related to the induction of GST activity. Further experiments are needed to determine whether or not *K. mikimotoi* itself or its toxins, as exogenous compounds, can induce GST activity. Additionally, it can be inferred that ROS produced by *K. mikimotoi* are eliminated through the GPx cycle via the catalase system rather than through the GST conjugation pathway.

ROS induces cell damage and can cause DNA strand breaks [54]. The comet assay is a simple assay to measure DNA strand breaks in eukaryotic cells and has been used as a genotoxic biomarker of environmental pollution in fish and aquatic species [55]. In this study, the comet assay was used to confirm that *K. mikimotoi* can induce DNA damage in *P. major* (Figure 5). The extent of DNA damage reached its maximum level after 3 h of exposure and decreased after 24 h of exposure and 3 h of depuration, which suggests that the repair mechanism of *P. major* can reduce the damage caused by *K. mikimotoi*. These findings are consistent with the results of the antioxidant enzyme-related experiments, which suggest that *P. major* can activate defense mechanisms to cope with the oxidative stress induced by *K. mikimotoi*. The inconsistent levels observed at 24 h can be explained by potential fluctuations in ROS levels and antioxidant defense capacity over the course of the experiment. In general, most ROS are short-lived, and their steady-state levels are low, changing rapidly due to varying rates of generation, chemical reactions, and diffusion [56]. The sequential reactions in the antioxidant defense system may contribute to the variations in ROS content. Therefore, conducting additional analyses at multiple time points in a detailed time course is required to better understand the correlations between ROS levels and antioxidant defense responses.

In vitro experiments have shown that toxins produced by dinoflagellates can induce DNA damage [57]. Similarly, gymnocin-A [58] and gymnocin-B [59], toxic substances produced by *K. mikimotoi*, may also cause DNA damage. However, algal toxins were not detected in the tissues of oysters exposed to *K. brevis*, one of the most toxic *Karenia* species. Therefore, it is possible that fish death could be caused by non-toxin factors, such as physical gill injury or environmental stress factors such as direct attachment and hypoxia [44]. In fact, rainbow trout exposed to hypoxic and hyperoxic conditions for 5 h showed induced DNA single-strand breaks (DSSB) [60], and hypoxia in sheepshead minnows also caused increased DNA damage, similar to the findings of this study [61]. Another study showed that *C. polykrikoides*, which produces different kinds of toxic substances, induced similar levels of MDA and DNA damage following exposure to red sea bream [62].

Taken together, while these findings provide clear evidence of acute oxidative stress induced by *K. mikimotoi* exposure, it is crucial to consider the potential presence of toxins produced by the dinoflagellate in order to fully understand the underlying toxicity mechanisms. Previous research suggests that *K. mikimotoi* does not contain shellfish poisons [38,39], brevetoxins [16], or karlotoxin [40]. Despite our efforts to minimize the potential impact of additional or unknown toxins by rapidly exposing fish to concentrated *K. mikimotoi* in a fresh medium, we cannot discount the possibility, as hemolytic toxins are recognized as another potential inducer of toxicity [30,31,63]. Since we lack a precise analytical procedure for measuring these potential toxins, further research is warranted to address this limitation.

In addition, these findings imply that both gill tissues and blood samples from red sea bream could serve as valuable materials for investigating the potential impacts of HABs in marine fish. The gill antioxidant enzyme activity exhibited alterations after 24 h, whereas blood samples displayed rapid changes in ROS-related indicators within 3 h. Given the ease of sample preparation and the overall reaction rate observed in the enzyme assay, blood samples are preferable to gill tissue samples.

## 4. Conclusions

**Figure 7:**
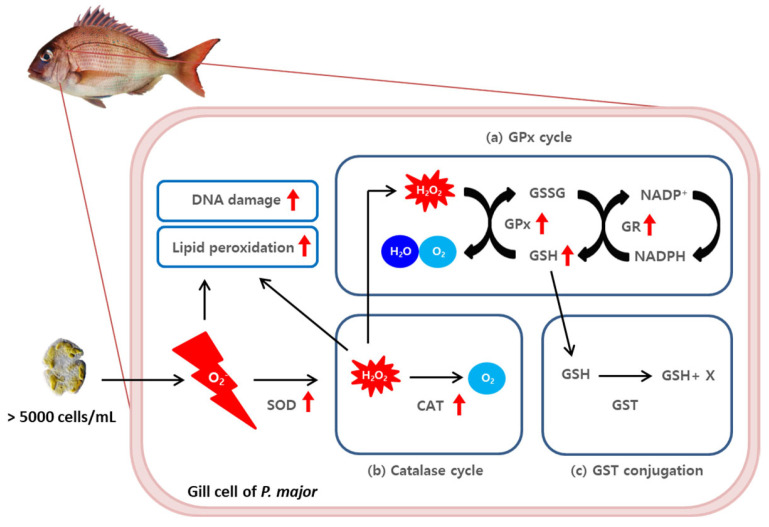
A schematic diagram of the ROS−induced (**a**) glutathione peroxidase cycle, (**b**) catalase cycle, and (**c**) GST conjugation scavenging pathway in the gill tissues of *P. major* after exposure to 5000 cells/mL of *K. mikimotoi*. The red arrow indicates a significant increased response (*p* value < 0.05).

This experiment aimed to investigate the mechanism underlying the toxicity of *K. mikimotoi* and to potentially reduce the economic losses of aquaculture caused by red tide disasters. The study found that exposure to high concentrations of *K. mikimotoi* led to the accumulation of ROS and oxidative DNA damage in the gill tissue of red sea bream. The increased activity of antioxidant enzymes, such as GPx, GR, CAT, and GSH, was observed to contribute to the elimination of intracellular ROS induced by *K. mikimotoi* exposure. These results support the hypothesis that increased antioxidant enzyme activity in the gill tissue of red sea bream protects the fish from oxidative stress induced by *Karenia* (Figure 7). Therefore, this study provides important insights into the potential mechanisms that protect fish from red tide toxicity and may contribute to the development of effective strategies (e.g., systematic early signal effectuation for HAB; the establishment of actionable knowledge in the fields of HAB) for mitigating the harmful effects of red tides on aquaculture.

## 5. Materials and Methods

### 5.1. Experimental Fish and K. mikimotoi Cultivation Conditions

All fish handling and experimental procedures were granted approval by the Animal Welfare Ethical Committee and the Animal Experimental Ethics Committee of Incheon National University (Incheon, Republic of Korea) and the Southeast Sea Fisheries Research Institute (Tongyeong, Republic of Korea). Healthy *P. major* red sea breams were used in this study and were obtained from an enclosure aquaculture facility in Tongyeong, Gyungnam, Republic of Korea. Individuals without any apparent disease symptoms were selected for testing. Juvenile (~6 months after hatching; total length, 15.44 ± 1.97 cm; body weight, 57.89 ± 5.47 g) red sea breams were selected for the study. The red sea breams were transferred from the enclosure aquaculture facility to the circulating aquaria and were kept in filtered seawater (32 psu) at 20 °C, with a 14 h:10 h light/dark cycle, under constant conditions. During the experiment, the fish were fed twice a day with either frozen mosquito larvae or an artificial diet until they were satiated before experimentation.

For the mass culture of *K. mikimotoi*, selenium (H_2_SeO_3_) was added at a final concentration of 0.001 μM in f/2 medium [64]. Seawater was sampled from the coast of Baegyado Bay in Yeosu at a salinity of 32 psu or higher and filtered via stepwise filtration using three types of filter paper with different pore sizes (1, 5, and 10 μm) using an analogous peristaltic pump (Masterflex, Gelsenkirchen, Germany). *K. mikimotoi* were cultured under 24 h of continuous light at 100 μmol photons/m^2^/s and 24–26 °C in 20 L Nalgene polycarbonate bottles or glass culture containers. All the experimental instruments were sterilized via autoclaving or sterilization to prevent secondary biological contamination.

### 5.2. Exposure to K. mikimotoi and Sample Preparation

*K. mikimotoi* was harvested for the exposure experiment between its exponential and early stationary phases to achieve final concentrations equivalent to ≈14,000 *K. mikimotoi* cells/mL. Cell numbers were counted with an inverted microscope (Nikon Eclipse TS 100; Tokyo, Japan) using Sedgewick-Rafter Counting Chamber (Graticules Ltd., Tonbridge, UK) with 0.1–1 mL of Lugol’s solution (2%).

Juvenile red sea breams were exposed to various concentrations of *K. mikimotoi* to investigate its molecular and biochemical effects on *P. major*. An experimental round tank with a capacity of 450 L was prepared for housing the fish during the exposure. To minimize potential stress, the fish were reared individually in the experimental tanks for five days prior to the exposure studies. In each concentration, 60 fish were individually reared in the tank, with 15 fish randomly grouped for each time course. Depuration was conducted by completely replacing the total exposure water with filtered seawater. The flow rate of the filtered seawater was set to 3 L per h, and water flow was monitored using electronic flow meters (TRACER^®^ Electronic Flowmeter, Grandview, MO, USA). Fifteen fish were collected at 0, 3, and 24 h, and after 3 h of depuration. The 15 collected fish were then randomly separated into three subgroups, each in triplicate (*N* = 5 for each subgroup). During the exposure, the fish were not fed.

For the collection of gill tissues from three subgroups, red sea breams exposed to *K. mikimotoi* were anesthetized with MS-222 solution (200 mg/L tricaine methanesulfonate, Sigma-Aldrich, St. Louis, MO, USA). Gill samples were obtained by dissecting each fish, and gill tissues weighing 32 ± 2.7 mg were collected. Blood samples from the remaining subgroups were collected using a heparin syringe. A 10 μL sample from each fish was mixed with 1 mL of cold fetal bovine serum (FBS; Gibco, MA, USA) and stored at 4 °C, wrapped with foil to prevent DNA damage due to light. For the analysis, five gill samples from each subgroup (*N* = 5) were pooled, and the two pooled samples were analyzed independently in triplicate. Additionally, five blood samples from each subgroup (*N* = 5) were analyzed separately. The detailed experimental method is presented in Supplementary Figure S1.

### 5.3. Measurements of Lipid Peroxidation Marker

Malondialdehyde (MDA) was measured to assess lipid peroxidation (LPO) in the red sea bream. Pooled gill tissues were homogenized by adding five volumes of buffer (20 mM Tris, 150 mM NaCl, 10 mM β-mercaptoethanol, 20 μM leupeptin, 2 μM aprotinin, and 100 μM benzamidine). All other reagents used in the experiments were GR-grade. The supernatants were collected after centrifugation at 30,000× *g* for 30 min at 4 °C, followed by denaturation for 15 min at 75 °C. To measure Thiobarbituric acid reactive substances (TBARS), malondialdehyde bis(dimethyl acetal) (MDA, Sigma-Aldrich) was used as the standard, and the absorbance was measured at 535 nm using a Varioskan Flash spectrophotometer (Thermo Fisher Scientific, Tewksbury, MA, USA). The results are reported as nM of MDA per gram of gill tissue.

### 5.4. Measurements of Antioxidant Molecule

To determine the concentration of intracellular GSH in pooled gill tissues, Glutathione Assay Kit (Catalog No. CS0260; Sigma-Aldrich) was used. Gill tissues were washed with 0.9% NaCl and then homogenized in trichloroacetic acid using a Teflon homogenizer at a ratio of 1:20 (*w*/*v*). The homogenate was centrifuged at 3000× *g* for 10 min at 4 °C, and the resulting supernatant was collected for the GSH assay. The measurement of GSH content was carried out following the manufacturer’s instructions and using a spectrophotometer to measure absorbance at 420 nm. 

### 5.5. Enzyme Activity Analysis

To assess the activities of enzymes associated with antioxidant defense mechanisms, such as catalase (CAT), superoxide dismutase (SOD), glutathione peroxidase (GPx), glutathione reductase (GR), and glutathione *S*-transferase (GST), after exposure to *K. mikimotoi* at different concentrations (1000, 3000, 5000, and 7000 cells/mL) and exposure times (0, 3, and 24 h; 3 h depuration), the gill tissue samples were collected and processed using slight modifications of a previously published protocol [65]. Total protein content was measured using the Bradford assay [66].

For CAT and SOD activities, the gill tissues were homogenized in ice-cold buffer (0.25 M sucrose, 0.5% Triton X-100, pH 7.5) using a Teflon homogenizer at a ratio of 1:4 (*w*/*v*). The homogenates were centrifuged at 3000× *g* for 30 min at 4 °C, and the upper layer containing the enzyme was used for the enzymatic assay according to the manufacturer’s instructions. The total activities of CAT and SOD were measured at 440 and 520 nm, respectively, using commercially available assay kits (Catalog No. CAT100 and 19160; Sigma-Aldrich).

For GPx and GR activities, the pooled samples were homogenized in cold buffer (50 mM Tris-Cl, 5 mM EDTA, and 1 mM β-mercaptoethanol, pH 7.5) at a ratio of 1:4 (*w*/*v*) using a Teflon homogenizer. The homogenate was centrifuged at 10,000× *g* for 10 min at 4 °C, and the upper aqueous layer containing the enzyme was collected for enzymatic assays using commercially available assay kits (Catalog No. CGP1 and GRSA; Sigma-Aldrich). The GPx and GR activities were measured at an absorbance of 340 nm.

To determine GST enzyme activity, the pooled gill tissues were homogenized in cold buffer (0.25 M sucrose, 10 mM Tris, 1 mM ethylenediaminetetraacetic acid (EDTA), 0.2 mM dithiothreitol [DTT], and 0.1 mM phenylmethylsulfonyl fluoride (PMSF), pH 7.4) at a ratio of 1:4 (*w*/*v*) using a Teflon homogenizer. The cytosolic fraction containing the enzyme was collected after centrifugation at 10,000× *g* for 10 min at 4 °C, and the enzymatic assay was performed using CDNB as the substrate. The conjugation of CDNB and GSH was monitored at 340 nm, and the enzymatic activities were normalized to the total protein and presented as a percentage of the control. 

### 5.6. Comet Assay

The alkaline comet assay was conducted with some adjustments as previously described [43]. In this method, 30 μL of the diluted sample mixed in 200 μL of 0.5% low-melting-point agarose (A9414, Sigma-Aldrich St Louis, USA) was prepared. Subsequently, 100 μL of this mixture was placed on pre-coated slides with 1% normal-melting-point agarose (Agarose A; Bio basic Inc., Markham, ON, Canada). The slides were then immediately covered with coverslips (Marienfeld, Germany) and kept in a refrigerator for 10 min to solidify. After removing the coverslips, a third layer containing 100 μL of 0.5% low-melting-point agarose was added to the slides and covered with coverslips again. The gel was then solidified, and the coverslips were removed. The slides were subjected to a cold lysis solution (2.5 M NaCl (Sigma-Aldrich); 100 mM Na_2_-EDTA (Sigma-Aldrich), 10 mM Tris (Sigma-Aldrich) at pH 10, supplemented with 10% DMSO (Ducksan Co., Gwangju, Republic of Korea) and 1% Triton X-100 (Sigma-Aldrich)), refrigerated at 4 °C for 2 h, and then the lysis buffer was removed. The slides were arranged side-by-side on a horizontal electrophoresis box and filled with fresh unwinding buffer (1 mM Na_2_-EDTA and 300 mM NaOH (Sigma-Aldrich), pH 13.5). The slides were left in the buffer for 20 min to allow for unwinding. Electrophoresis was conducted using the same buffer at 25 V and 300 mA for 25 min. The electrophoresed slide was washed thrice for 10 min in 0.4 M Tris buffer. Dehydration was then carried out in 70% ethanol, dried, stained with 75 μL ethidium bromide (EtBr; Sigma-Aldrich) (20 μg/mL), and examined with a fluorescence microscope equipped with the appropriate filters. In total, 100 cells (50 per replicate) per sample (*N* = 5) were randomly scored at a 400 × magnification and analyzed using the Komet 6.0 image analyzer system (Kinetic Imaging, Nottingham, UK). The Olive tail moment was the comet parameter used to quantify DNA damage, which was calculated using the software (Tail Moment = Tail DNA% × Tail Moment Length).

### 5.7. Statistical Analyses

Statistical analyses were conducted using GraphPad Prism software version 10.0.2 for Windows (GraphPad Software, Boston, MA, USA). The data were assessed for normality using the Shapiro–Wilk test. Bartlett’s test confirmed that the data did not exhibit equal variances (homoscedasticity). Consequently, we employed Welch’s one-way analysis of variance (ANOVA) to accommodate data with unequal variances. For post hoc analysis, we utilized Dunnett’s T3 test to assess differences between the exposure groups and the control group. Pearson’s correlation analysis was performed to determine the relationships among various variables. *p* values less than 0.05 were considered statistically significant. All statistical data are expressed as mean ± standard deviation. 

## Figures and Tables

**Table 1:** Status of *Karenia mikimotoi* blooms in Republic of Korea from 2014 to 2022.

Year	2014	2015	2016	2017~2022
Initiation	11 August	4 August	2 August	No occurrence
Termination	22 August	2 September	26 August	-
Duration (d)	12	30	25	-
Affected area	Goseong	Yeosu, Goseong, Tongyeong, Busan	Yeosu	-
Max. density (cells/mL)	2000	9000	2200	-
Water temperature (°C)	24.2~25.9	22.7~26.7	24.7~31.1	-

The data were collected by gathering the information from the red tide monitoring program provided by NFRDI. No *Karenia mikimotoi* bloom occurred from 2017 to 2022.

## Data Availability

Not applicable.

## Supplementary Materials

The following supporting information can be downloaded at: https://www.mdpi.com/article/10.3390/toxins15100620/s1, Figure S1: The detailed experimental protocol.
