# Supplementary figures and images for: Oxidative Stress and DNA Damage in Pagrus major by the Dinoflagellate Karenia mikimotoi
Source: Toxins (Basel). 2023 Oct 19;15(10):620. doi: 10.3390/toxins15100620 (PMC10611101; doi:10.3390/toxins15100620)

## Supplementary Figure S1

### The detailed experimental protocol

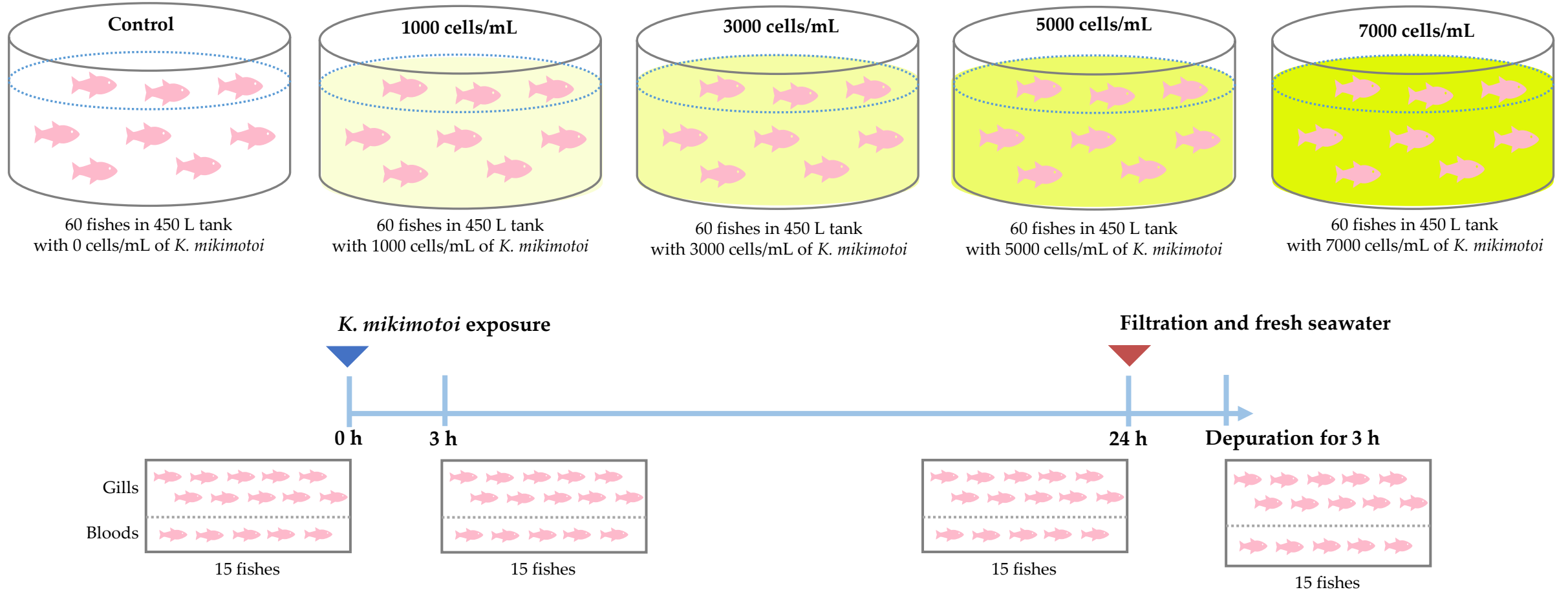

Supplement: Supplementary file 1 [file toxins-15-00620-s001.zip › toxins-2271478-supplementary.pdf]
